# Identification of serum prognostic marker miRNAs and construction of microRNA-mRNA networks of esophageal cancer

**DOI:** 10.1371/journal.pone.0255479

**Published:** 2021-07-30

**Authors:** Yue Jiang, Chengda Zhang, Wenbin Shen, Yiming Li, Yun Wang, Jianjun Han, Tao Liu, Li Jia, Fei Gao, Xiaojun Liu, Mi Chen, Guangming Yi, Hongchun Dai, Jun He

**Affiliations:** 1 Department of Clinical Medicine, Southwest Medical University, Luzhou, China; 2 Department of Gastroenterology, The Third Hospital of Mian Yang (Sichuan Mental Health Center), Mianyang, China; 3 Department of Oncology, The Third Hospital of Mianyang (Sichuan Mental Health Center), Mianyang, China; 4 Department of Oncology, The First Affiliated Hospital of Chengdu Medical College, Chengdu, China; 5 The Third Hospital of Mianyang (Sichuan Mental Health Center), Mianyang, China; University of Ulsan College of Medicine, REPUBLIC OF KOREA

## Abstract

Esophageal cancer is a common tumor of the digestive system with poor prognosis. This study was to gain a better understanding of the mechanisms involved in esophageal cancer and to identify new prognostic markers. We downloaded the esophageal cancer miRNA expression profile microarray data (GSE113740, GSE112264, GSE122497, GSE113486, and GSE106817) from the GEO database, extracted the esophageal cancer miRNA sequencing data from The Cancer Genome Atlas (TCGA) database, and then used a bioinformatics approach to select common differentially expressed miRNAs (DEMs). Differentially expressed genes (DEGs) were selected by predicting DEM target genes using the miRWalk database and intersecting with differential genes obtained from TCGA database for esophageal cancer. The STRING database was used to obtain protein–protein interaction (PPI) relationships to construct the DEM-DEG network. Furthermore, we selected core genes and core miRNAs associated with esophageal cancer prognosis by performing survival and univariate/multivariate COX analysis on DEMs and DEGs in the network and performed GSEA analysis on core genes alone, and finally the expression of the markers was verified by qPCR in esophageal cancer cell lines Eca109, SKGT-4 and normal esophageal epithelial cells HEEC. Nine DEMs were obtained, of which three were upregulated and six were downregulated, and 326 DEGs were obtained, of which 105 were upregulated and 221 were downregulated. Survival univariate/multivariate COX analysis revealed that five genes, ZBTB16, AQP4, ADCYAP1R1, PDGFD, and VIPR2, and two microRNAs, miR-99a-5p, and miR-508-5p, were related to esophageal cancer prognosis. GSEA analysis showed that the following genes may be involved in esophageal cancer prognosis: ZBTB16 may through the MTOR signaling pathway, AQP4 through the GNRH signaling pathway, ADCYAP1R1 through the PPAR signaling pathway, VIPR2 through the P53 signaling pathway and PDGFD through the PENTOSE-PHOSPHATE signaling pathway.

## Introduction

Esophageal cancer is a joint malignant tumor of the digestive system, primarily including two types of squamous carcinoma and adenocarcinoma [1, 2]. Worldwide, approximately 500,000 patients are diagnosed with esophageal cancer each year, and their 5-year overall survival rate is generally <15% [3, 4]. Although esophageal cancer treatment has greatly improved the prognosis of patients, the prognosis of patients with esophageal cancer remains poor as endoscopic diagnosis of early stage esophageal cancer is difficult and most patients with esophageal cancer are already in the middle or advanced stages at the time of diagnosis [5–7]. MicroRNAs (miRNAs) are small noncoding RNAs that regulate gene expression and are implicated in the pathogenesis of several cancers [8–10]. It has been shown that measuring circulating miRNA levels in patients’ serum may be a simple and non-invasive method for diagnosing certain early-stage cancers [11–13]. In the present study, to explore the mechanism underlying esophageal cancer development and provide novel targets and pathways for esophageal cancer diagnosis and treatment, a bioinformatics approach was used to analyze the esophageal cancer data derived from the public databases The Cancer Genome Atlas (TCGA) [14] and Gene Expression Omnibus (GEO) [15]. The differential genes and differential miRNAs of esophageal cancer were integrated and analyzed to construct a miRNA-mRNA network for esophageal cancer, and survival analysis, univariate/multivariate COX analysis, and GSEA analysis were conducted on the genes and miRNAs in the network to provide research directions for further basic experiments.

## Materials and methods

### Data

The GEO (https://www.ncbi.nlm.nih.gov./geo/) [15] database and TCGA (https://portal.gdc.cancer.gov/) [14] database provide a large amount of data on patients with esophageal cancer. In the GEO search box, we entered “esophageal cancer serum” and “miRNA” and selected human microarray data with sample size greater than 100 and then selected five datasets, GSE113740 [16], GSE112264 [17], GSE122497 [18], GSE113486 [19], and GSE106817 [20], from the same platform GPL21263 (3D-Gene Human miRNA V21_1.0.0), as shown in Table 1, including the serum miRNA microarray data from a total of 5869 healthy controls and 769 patients with esophageal cancer(Exclusion of large deviations from the data set GSE124158). RNA sequencing data (miRNA and mRNA) derived from the tumor tissues of patients with esophageal cancer and tissues of healthy controls were downloaded from TCGA database, select TCGA-ESCA’s sequencing reads date, including the miRNA sequencing data from 13 healthy tissues and 185 tumor tissues versus the mRNA sequencing data from 11 healthy tissues and 160 tumor tissues.

**Table 1:** Grouping characteristics of each data set.

Date Set	Contributors	Samples of EC	Samples of HC	Submission date
**GSE113740 [13]**	Yamamoto Y, et al	25	969	30-Jul-20
**GSE112264 [14]**	Urabe F, et al	50	41	Mar 23,2018
**GSE122497 [15]**	Sudo K, et al	566	2000 (Select the first two thousand)	13-Nov-18
**GSE113486 [16]**	Usuba W, et al	40	100	20-Apr-18
**GSE106817 [17]**	Yokoi A, et al	88	2759	13-Nov-17

EC: Esophageal Cancer; HC: healthy control.

Human esophageal cancer SKGT-4 and ECA109 cells, human normal esophageal epithelial cells (HEEC cells) were purchased from the Cell Bank of the Chinese Academy of Sciences; fetal bovine serum and RPMI1640 cell culture medium were purchased from Hyclone; RNA extraction kit, Trizol and protein extraction lysis solution were purchased from Biyuntian Biotechnology Company Limited.

### Difference analysis

The data in the five datasets were divided into cancer and healthy control groups, and the results were analyzed differentially and visualized using the GEO2R tool based on the limma.R package (https://www.ncbi.nlm.nih.gov./geo/geo2r/) [21], setting padj = 0.05 and log2FC = 1. The results were incorporated using the RobustRankAggreg.R [22] package, and an R package for the meta-analysis of multiple sets of GSE data was used to integrate the differential results and select differentially expressed miRNAs with p < 0.05 (DEMs1). Differential analysis of data obtained from TCGA database was performed using the Edge.R [23] package, and to ensure the accuracy of the results, read counts were transformed into counts per million index to eliminate the effect of sequencing depth. Differential miRNAs with Q values < 0.05, log2FC > 1 were selected as DEMs2 and Q values < 0.05, log2FC > 2 were used as initial differentially expressed genes (DEGs). DEMs1 and DEMs2 were selected for intersection to obtain differentially expressed miRNAs (DEMs).

### miRNA-mRNA and PPI network analysis

The miRWalk database [24] and the STRING database [25] were used for the construction of the miRNA-mRNA network in esophageal cancer. A total of 326 DEGs were obtained by predicting the target genes of DEMs using the miRWalk website and taking intersections with the abovementioned DEGs. The STRING database contains several protein–protein interaction relationships, and the protein–protein association (PPI) of 326 DEGs was analyzed using the STRING database. The DEGs with a combined score > 0.7 (139 genes in total) were selected and outlined with their upstream miRNAs to create an esophageal cancer miRNA-mRNA network using the Cytoscape [26] software to visualize the results.

### Univariate/Multivariate COX analysis

Univariate / Multivariate COX analysis and survival analysis often establishes a link with disease prognosis and is widely used in oncology research. We used genome-wide clinical data downloaded from TCGA for 160 patients with esophageal cancer to initially screen 139 genes in the network using univariate COX analysis, before further screening esophageal cancer prognostic association DGEs using multivariate COX analysis and constructing a prognostic model for esophageal cancer to test the prognostic model effect using survival curves and ROC curves. Only DEGs that met p<0.05 for both univariate COX and multivariate COX analyses could be considered as prognostic-associated DEGs for esophageal cancer. Multivariate COX analysis was performed online for DEMs using the OncomiR database [27], and then p<0.05 Kaplan-Meier survival curves were plotted for DEMs based on the high and low risk values obtained from the COX analysis.

### GSEA analysis of core genes

Gene set enrichment analysis (GSEA) analysis can be used for the pathway enrichment analysis of individual genes, which can better reveal the upregulation and downregulation relationships of individual gene-enriched pathways in disease compared with the common KEGG analysis. The 160 esophageal cancer samples were divided into gene-high and gene-low expression groups, and all esophageal cancer genes were analyzed for enrichment in GSEA software [28], and some pathways with p-values <0.05 were selected for visualisation. The pathways that were predominantly enriched for genes were examined in preparation for the subsequent basic experiments.

### Cell culture and qPCR

Human esophageal cancer SKGT-4, ECA109 cell lines and HEEC cell lines were cultured in sterile RPMI1640 medium containing 10% fetal bovine serum and 1% antibacterial drugs (penicillin, streptomycin) in a sterile cell culture incubator at 37°C and 5% CO2.

RT-qPCR is a sensitive technique for quantifying specific RNA targets. Human esophageal cancer SKGT-4 and ECA109 cells at logarithmic growth stage were collected, with HEEC cells as control. RNA was extracted using the RNA extraction kit, strictly according to the instructions. RNA concentration was detected by qPCR. cDNA was synthesized by reverse transcription and PCR reactions (PCR reaction conditions: 95°C30s,95°C10s,60°C30s) were performed to detect the expression levels of miR-99a-5p, miR-508-5p, ZBTB16, AQP4, ADCYAP1R1, PDGFD and VIPR2. The relative expression levels of the genes were calculated as 2^-ΔΔCt^.

## Results and discussion

### Differentially expressed miRNAs (DEMs)

**Fig 1:**
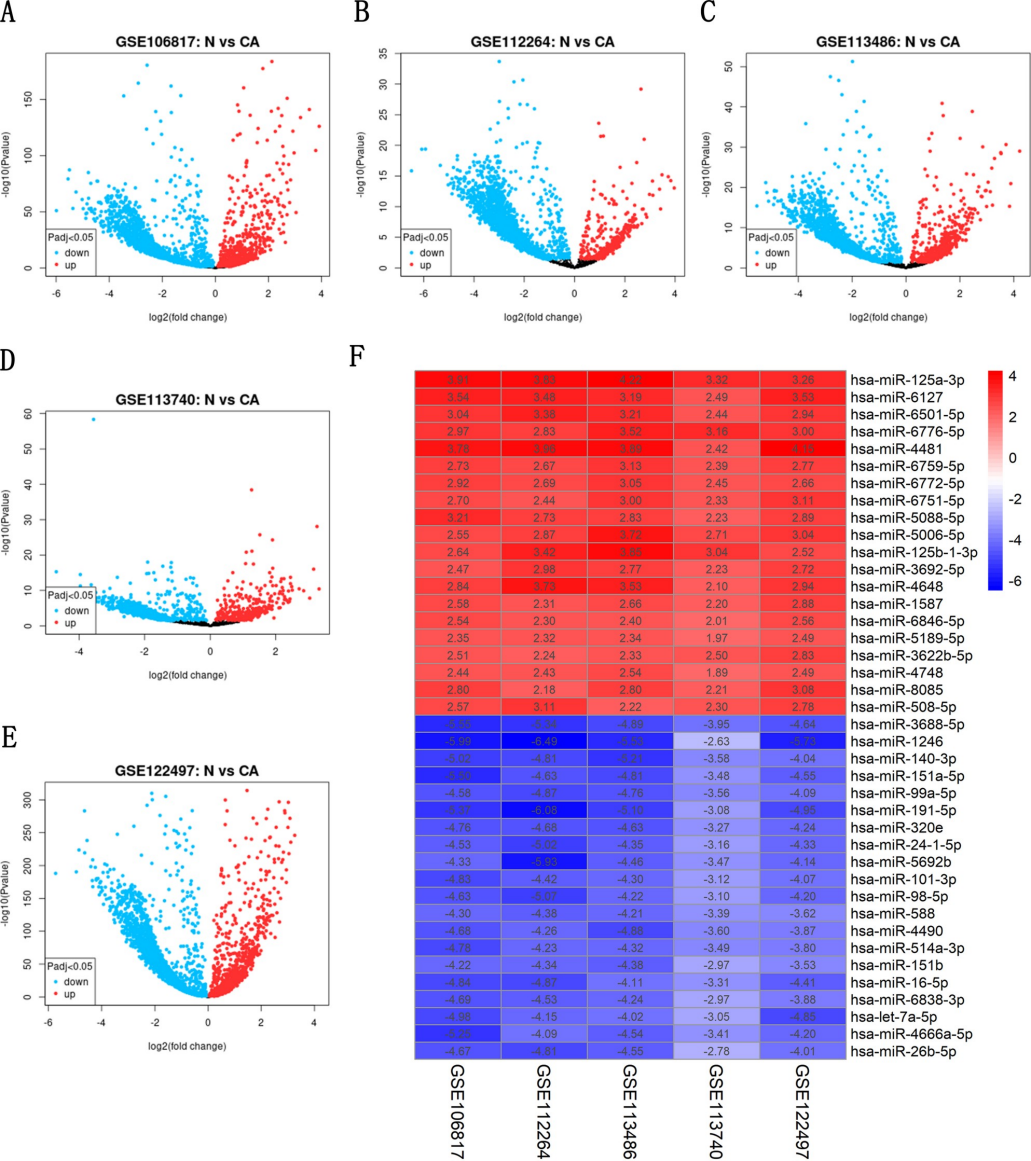
Volcano plots of differentially expressed genes in GSE113740, GSE112264, GSE122497, GSE113486, and GSE10681 and log2FC heatmap of each expression microarray. (A-E) show the volcano plots of differentially expressed genes in GSE10681, GSE112264, GSE113486, GSE113740, and GSE122497, respectively. The horizontal coordinates represent log2 (fold change), blue represents downregulated genes, and red represents upregulated genes. The vertical coordinate represents -log10 (p value). N is the normal control group; CA is the cancer group. (F) shows the log2FC heat map of GSE113740, GSE112264, GSE122497, GSE113486, and GSE10681. The numbers in the plot show the log2FC for each expression microarray, with red indicating upregulation, and blue indicating downregulation.

**Fig 2:**
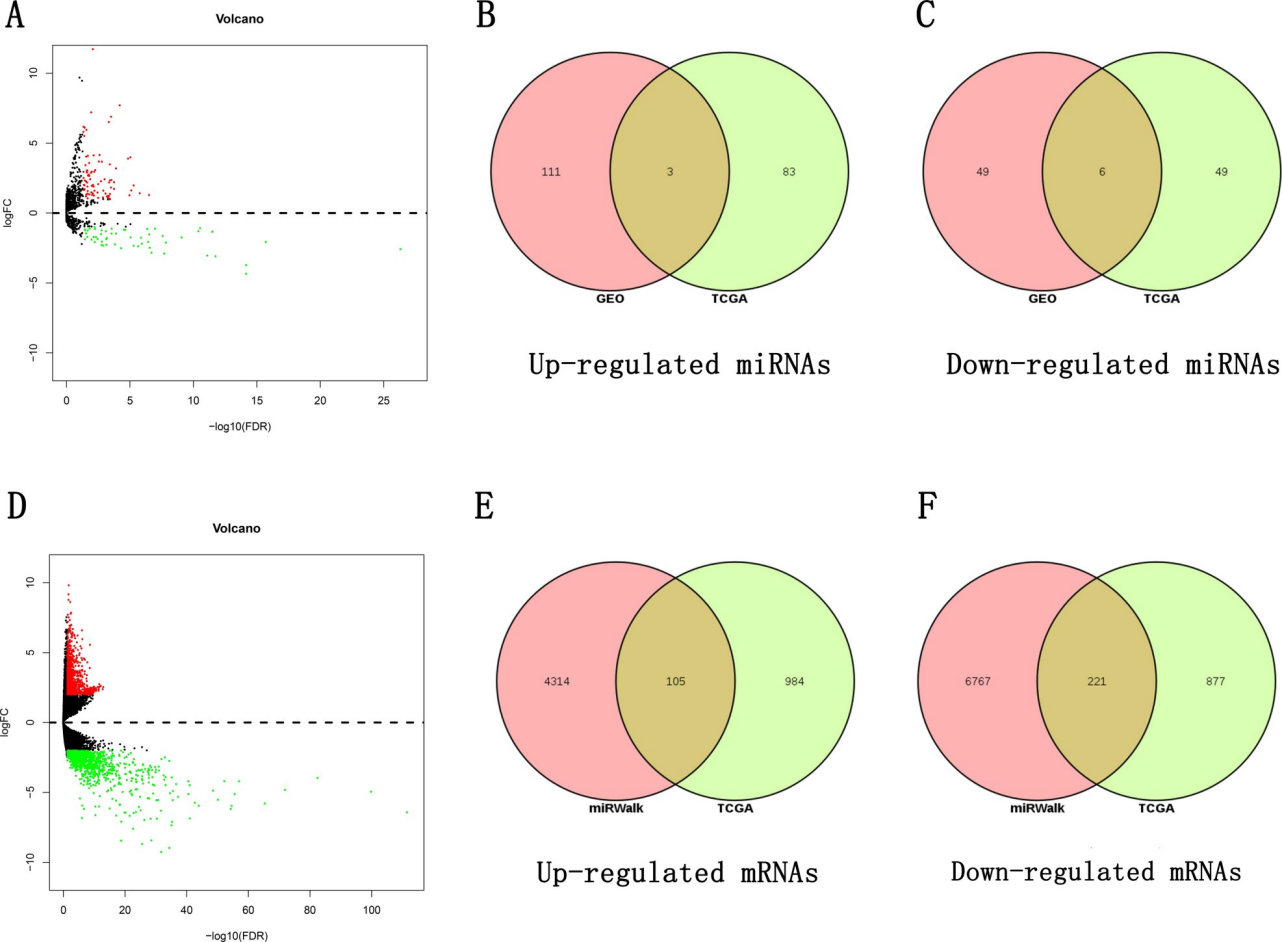
Differentially expressed miRNAs (DEMs) and differentially expressed genes (DEGs). (A) and (D) indicate the differential volcano plots of differentially expressed miRNAs in esophageal cancer tissues versus normal tissues in TCGA database and differential volcano plots of differentially expressed genes in esophageal cancer tissues versus normal tissues in TCGA database, respectively, where red indicates upregulation, and green indicates downregulation. (B) and (C) indicate the intersecting Venn diagrams of upregulated DEM1 and upregulated DEM2 and the intersecting Venn diagrams of downregulated DEM1 and downregulated DEM2, respectively. (E) and (F) indicate the Wayne diagrams of intersection of DEMs predicted upregulated target genes and TCGA database differentially expressed upregulated genes and the Wayne diagrams of intersection of DEMs predicted downregulated target genes and TCGA database differentially expressed downregulated genes, respectively.

To obtain differential miRNAs for esophageal cancer, we conducted the differential analysis of sequencing data using the GEO2R tool to obtain differential volcano plots (Fig 1A–1E). The results of all differential analyses were analyzed using the RobustRankAggreg.R package, and miRNAs with p values < 0.05 were selected as DEMs1, after which the results were visualized (Fig 1F). Differential analysis of miRNA sequencing data derived from 13 healthy tissues and 185 tumor tissues in TCGA database was performed using the Edge.R package, and a differential volcano plot was obtained (Fig 2A). The differential miRNAs with p < 0.05 and log2FC > 1 were selected as DEMs2, and the final DEMs were obtained by taking the intersection of DEMs1 and DEMs2 (Fig 2B and 2C). A total of 169 DEMs1 and 141 DEMs2 were obtained, resulting in 9 DEMs, of which 3 were upregulated and 6 were downregulated.

### Differentially expressed genes (DEGs)

To obtain differential genes for esophageal cancer, we used the Edge.R package to perform the differential analysis of mRNA sequencing data derived from 11 healthy and 160 tumor tissues downloaded from TCGA database to obtain a differential volcano plot (Fig 2D), and the gene with p < 0.05 and log2FC > 2 was selected as the initial differential gene (DEGs1). The miRWalk database was used to predict the target genes of DEMs, all possible target genes were obtained, and the final DEGs were obtained by intersecting DEGs1 with the target genes (Fig 2E and 2F). A total of 326 DEGs were obtained, of which 105 were upregulated and 221 were downregulated.

### miRNA-mRNA and PPI networks

**Fig 3:**
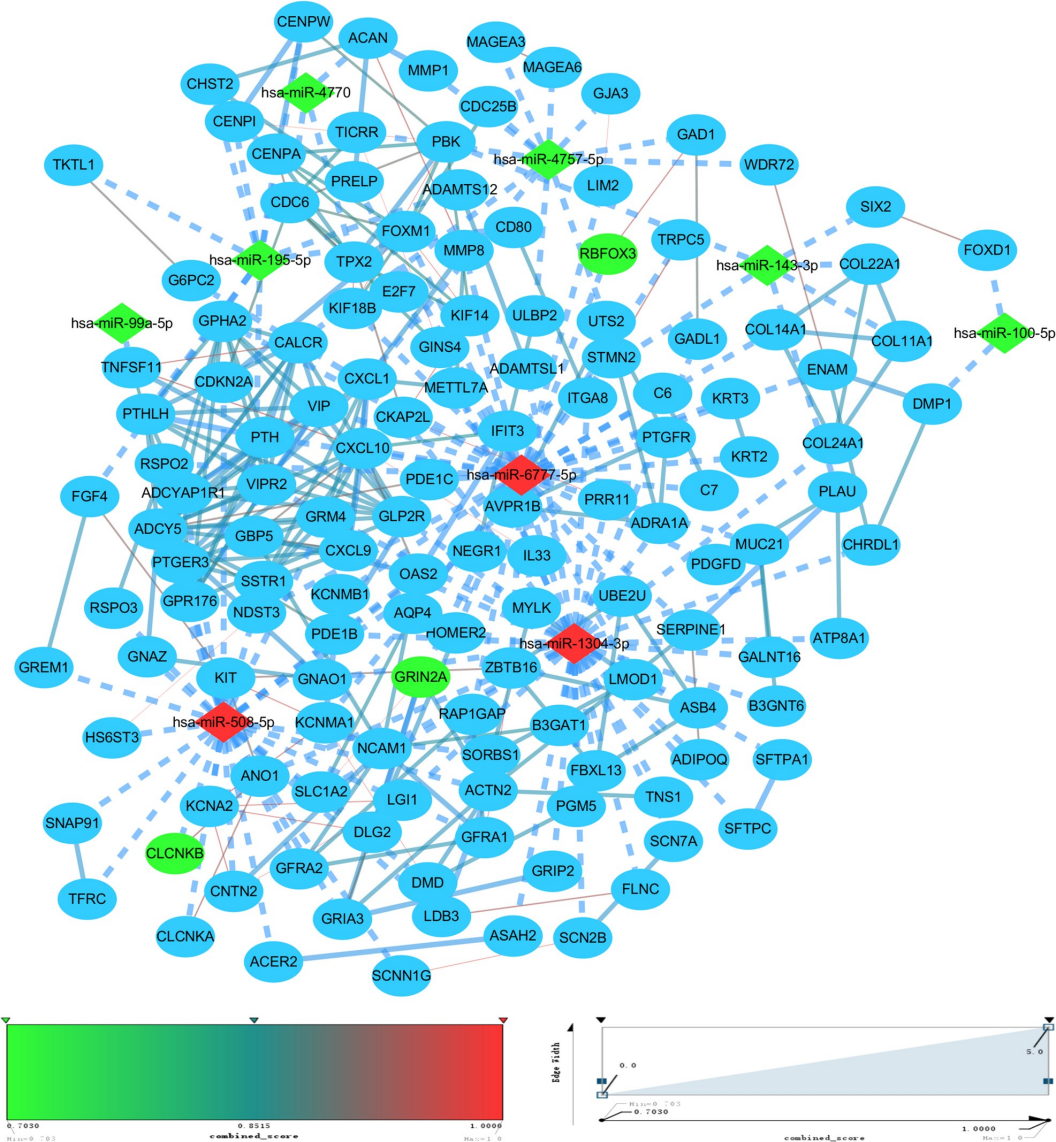
Diagram of miRNA-mRNA network in esophageal cancer and PPI network of DEGs. The above miRNA-mRNA network is based on the miRWalk database, where miRNAs are represented by diamonds, and genes are represented by circles. PPI linkage of DEGs was obtained by STRING database screening, where red indicates upregulation in esophageal cancer, green indicates downregulation, marquee dash-type lines indicate miRNA–gene interactions, and solid lines indicate gene-to-gene interactions. The color and thickness of the lines in the PPI network of DEG are drawn according to the combined score from the SRTING database.

To construct the miRNA-mRNA network for esophageal cancer and discover key markers of esophageal cancer from multiple perspectives, we submitted the DEGs to the STRING database for protein–protein interaction (PPI) analysis, identified the gene associations from the protein level, selected genes with stronger associations, and visualized them using the Cytoscape software (Fig 3).

### Univariate / Multivariate COX analysis

**Fig 4:**
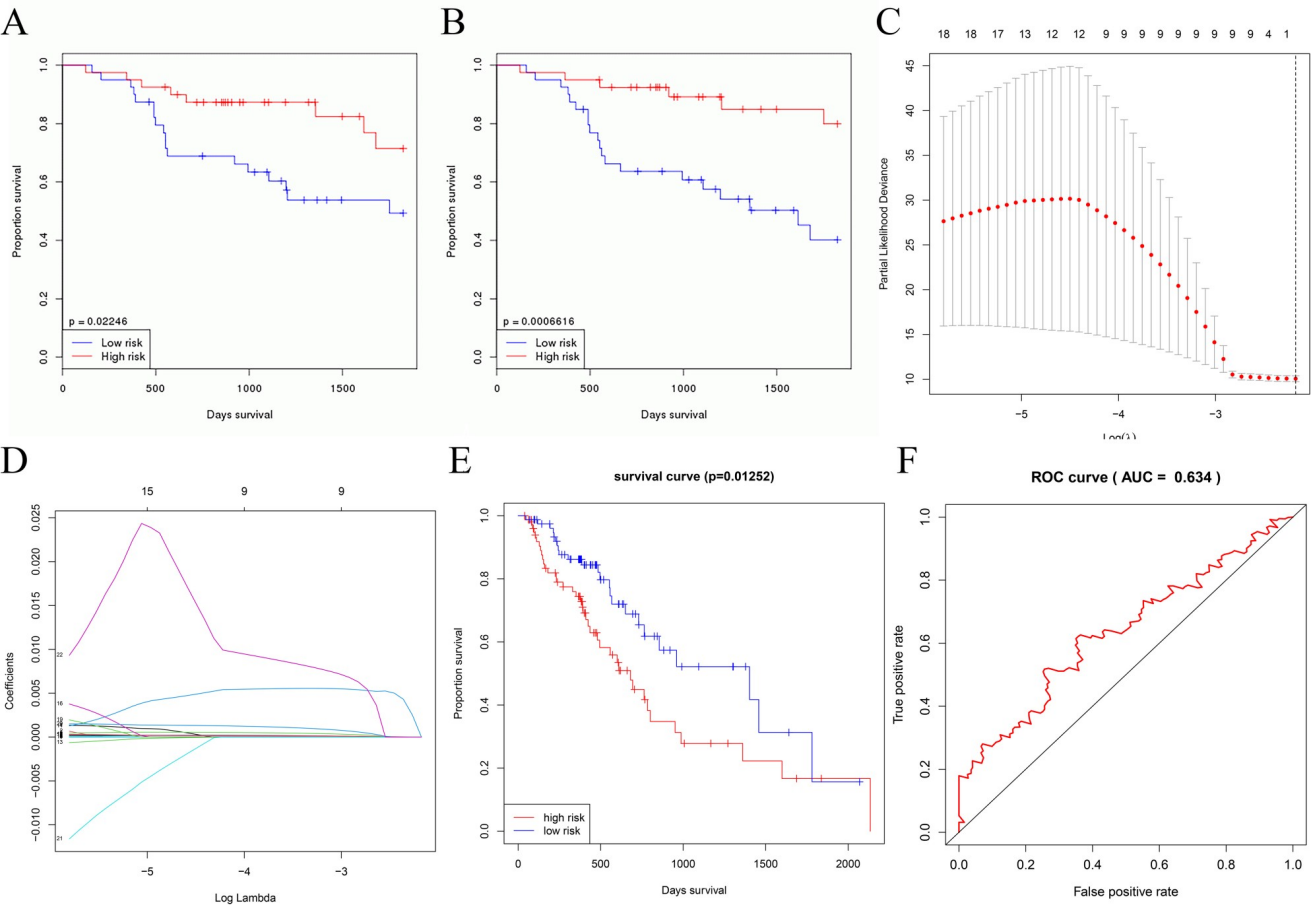
Prognostic risk models for ZBTB16, AQP4, ADCYAP1R1, PDGFD, VIPR2 and survival curves for miR-99a-5p and miR-508-5p. (A) and (B) indicate the survival curves of miR-99a-5p and miR-508-5p, respectively. The higher the expression of miR-99a-5p and miR-508-5p in esophageal cancer, the better the prognosis of patients. (C-F) indicate the cvfit plot, lambda plot, survival curve and ROC curve of the LASSO regression of the genetic prognostic model, respectively.

To obtain further core genes and core miRNAs associated with the prognosis of esophageal cancer, this study used clinical data from 11 healthy patients and 160 esophageal cancer patients in the TCGA database to perform univariate/multivariate COX analysis on 139 DEGs in the network, and univariate COX analysis yielded 22 prognosis-associated genes. Multivariate COX analysis yielded SIX2, ADIPOQ, ZBTB16, AQP4, ADCYAP1R1, PDGFD and VIPR2 seven (of which p<0.05 for a total of five genes ZBTB16, AQP4, ADCYAP1R1, PDGFD and VIPR2) genes associated with prognosis in esophageal cancer, seven-gene prognostic model survival p = 0.01252, area under the ROC curve AUC = 0.634 (Fig 4C–4F). OncomiR, a powerful database for studying cancer miRNAs online, was used to perform multivariate COX survival analysis of DEMs using 184 esophageal cancer clinical data in OncomiR, and miRNAs with p<0.05 were selected as the last associated with esophageal cancer prognosis of de novo markers (Fig 4A and 4B).

### GSEA analysis of core genes

**Fig 5:**
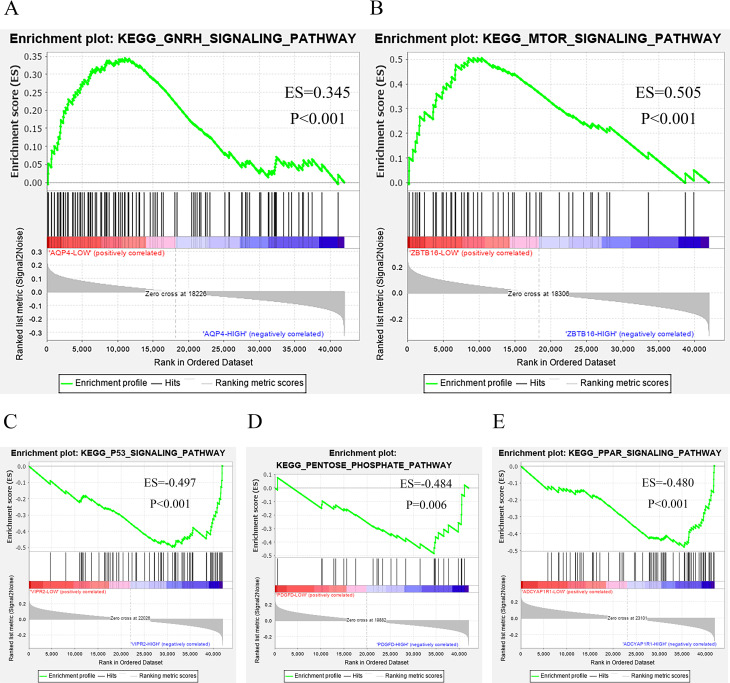
GSEA enrichment curves for ZBTB16, AQP4, ADCYAP1R1, PDGFD and VIPR2. (A-E) GSEA enrichment curves for AQP4, ZBTB16, VIPR2, PDGFD and ADCYAP1R1 based on gene-high and low expression groups using GSEA software, respectively. Where ES denotes Enrichment Score and p denotes Nominal p-value. (A) AQP4 probably through the GNRH signaling pathway, (B) ZBTB16 probably through the MTOR signaling pathway, (C) VIPR2 probably through the P53 signaling pathway, (D) PDGFD probably through the PENTOSE- PHOSPHATE signaling pathway and (E) ADCYAP1R1 may play a role in the prognosis of esophageal cancer through the PPAR signaling pathway.

The expression values of five genes, ZBTB16, AQP4, ADCYAP1R1, PDGFD and VIPR2, were sorted and divided into gene-high and low expression groups for patients with esophageal cancer, and then the core genes were sequenced using the esophageal cancer RNA downloaded from the TCGA database, and after counts per million (CPM) quantification, the GSEA software was used to The high- and low-expression groups were subjected to GSEA pathway enrichment analysis, and the top-ranked pathways with p value < 0.05 were selected for visualization, respectively (Fig 5A–5E).

### qPCR

**Fig 6:**
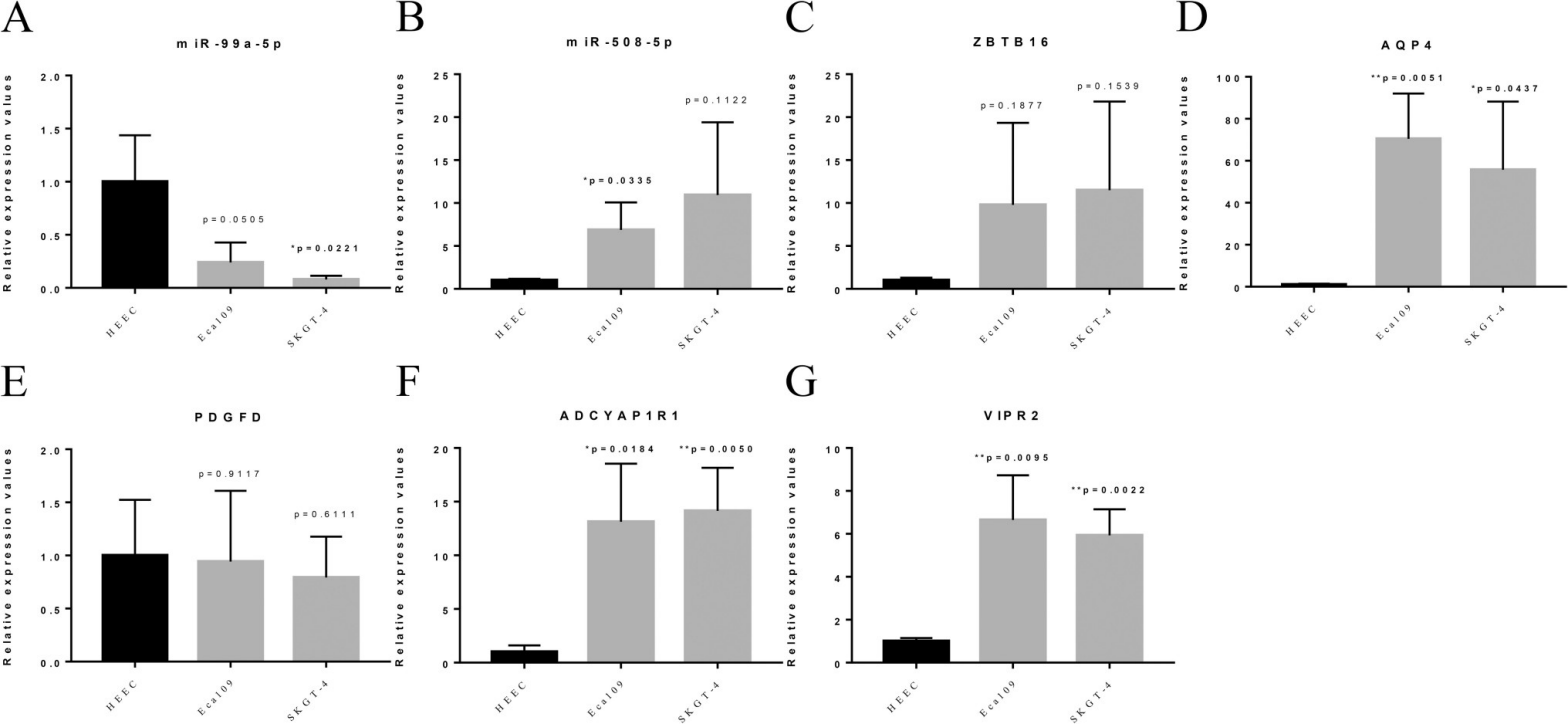
Relative expression of markers. (A-G) denote the expression of miR-99a-5p, miR-508-5p, ZBTB16, AQP4, PDGFD, ADCYAP1R1 and VIPR2, respectively. qPCR results showed that: (A) miR-99a-5p was significantly down-regulated in human esophageal cancer SKGT-4 cells compared with HEEC cells, and the difference was statistically significant. The differences were statistically significant. (B) The relative expression levels of miR-508-5p were significantly up-regulated in human esophageal cancer ECA109 cells compared with HEEC cells, and the differences were statistically significant. The relative expression levels of AQP4, ADCYAP1R1 and VIPR2 were significantly upregulated in human esophageal cancer SKGT-4 and ECA109 cells, and the differences were statistically significant.

The results showed that the relative expression levels of miR-508-5p, AQP4, ADCYAP1R1 and VIPR2 were significantly up-regulated in human esophageal cancer SKGT-4 and ECA109 cells compared with HEEC cells, and the differences were all statistically significant (Fig 6B, 6D, 6F and 6G). The relative expression levels of miR-99a-5p were significantly down-regulated in human esophageal cancer SKGT-4 cells compared with HEEC cells, and the differences were statistically significant (Fig 6A).

### Significance and impacts

The method of analyzing sequencing and microarray data through bioinformatics approaches is widely applied in the medical field, including the field of research on tumor mechanisms [29–31]. Bioinformatics can use an existing massive database to make predictions about unknown problems, which is a good solution to the challenge of how to screen out the core genes that are closely associated with diseases from a large number of genes. Esophageal cancer is one of the common tumors with poor prognosis, and according to current statistical results, China has the highest incidence of esophageal cancer in the world [32, 33]. To better screen people at high risk of esophageal cancer at an early stage, to improve the prognosis of esophageal cancer and to provide new directions for research on esophageal cancer, in the present study, we used data from the GEO database and TCGA database to analyze differentially expressed miRNAs in esophageal cancer and obtained a total of nine DEMs, of which three were upregulated and six were downregulated. We used sequencing data obtained from TCGA database to analyze DEGs in esophageal cancer and combined them with DEMs. The miRNA-mRNA network of esophageal cancer was constructed by combining the sequencing data with DEMs, which provides a new direction for research on esophageal cancer. To further understand the new biomarkers that are closely related to esophageal cancer prognosis, we also performed the univariate/multivariate COX analysis of miRNAs and mRNAs in the network and found five core genes, ZBTB16, AQP4, ADCYAP1R1, PDGFD and VIPR2, that may be associated with esophageal cancer prognosis and two core miRNAs, miR-99a-5p, and miR-508-5p. Finally, we conducted KEGG enrichment analysis on each of the five core genes using data obtained from TCGA database and GSEA software, expecting to identify the pathways acted upon by the core genes for subsequent basic experiments.

Zinc Finger And BTB Domain Containing 16 (ZBTB16) is a member of the Krueppel C2H2 type zinc finger protein family, which is associated with skeletal defects, genital hypoplasia, mental retardation and acute promyelocytic leukaemia [34–36]. Recent studies have demonstrated that ZBTB16 can act as an oncogene in breast cancer by upregulating ZBTB28 and antagonizing BCL6 [37]. Aquaporin 4 (AQP4), a member of the family of membrane protein water channel proteins, has been suggested to promote tumor progression, invasion and metastasis [38, 39] and is a promising target in oncology research. In lung squamous cell carcinoma, AQP4 is transcribed at low levels [40]. However, the role and mechanisms of ZBTB16 and AQP4 in esophageal cancer have not yet been investigated. Adenylate Cyclase Activating Polypeptide 1 (Pituitary) Receptor Type I (ADCYAP1R1) encodes a type I adenylate cyclase activating polypeptide receptor, which is associated with diseases such as post-traumatic stress disorder and regulatory spasticity [14, 15]. ADCYAP1R1 is differentially expressed in a variety of tumors, such as gliomas, breast and prostate cancers, and gastric cancer [41–43]. This also includes esophageal cancer, where Zhang, Yuefeng et al. found low expression of ADCYAP1R1 in esophageal cancer by bioinformatics analysis consistent with our experimental results [44], but the exact prognostic mechanism of ADCYAP1R1 affecting esophageal cancer has not been investigated. PDGFD (platelet-derived growth factor D) is a protein-coding gene, Yang, Xiao et al. demonstrated through bioinformatics that PDGFD is associated with ovarian cancer [45]. Although PDGFD has not been shown to be associated with prognosis in esophageal cancer, the association between platelet-derived growth factor-BB expression and prognosis in human esophageal squamous cell carcinoma has been studied [46]. VIPR2 encodes the receptor for vasoactive intestinal peptide (a small neuropeptide), which has been associated with diseases such as schizophrenia [47]. VIPR2 is under-expressed in ESCC, which is consistent with our experimental results [48].

## Conclusion

In conclusion, our study screened prognostic markers for esophageal cancer, constructed a miRNA-mRNA network for esophageal cancer, and predicted possible convergence pathways for core genes, laying the foundation for subsequent basic research on esophageal cancer. However, there are still shortcomings and limitations in our study. 1) We cross-analyzed the obtained serum differential DEMs with the tumor tissue DEMs in the TCGA database, which is a case of excluding miRNAs secreted by tumor tissue into serum, which may lead to the omission of some key miRNAs. To facilitate peer research on circulating miRNAs in esophageal cancer serum, we have published the results of the GEO analysis as a supplementary paper. (2) Our proposal of circulating miRNAs in serum as a possible new diagnostic marker is based on speculation from previous studies and theories, and the exact method of how to extract the corresponding miRNAs from serum needs to continue to be investigated. (3) Our experiments were validated using only one experimental method, qPRC, and two esophageal cancer cell lines, which likely led to biased results. The expression of each gene also appeared to be overexpressed. (4) The pathways and miRNA-mRNA networks were obtained by bioinformatics analysis and should be further validated by experiments.

## Supporting information

S1 Checklist (DOCX)
