# Correction: Identification of serum prognostic marker miRNAs and construction of microRNA-mRNA networks of esophageal cancer

**DOI:** 10.1371/journal.pone.0302499

**Published:** 2024-04-19

**Authors:** Yue Jiang, Chengda Zhang, Wenbin Shen, Yiming Li, Yun Wang, Jianjun Han, Tao Liu, Li Jia, Fei Gao, Xiaojun Liu, Mi Chen, Guangming Yi, Hongchun Dai, Jun He

There are errors in the author affiliations. The correct affiliations are as follows:

Yue Jiang^1^, Chengda Zhang^2^, Wenbin Shen^3^, Yiming Li^2^, Yun Wang^4^, Jianjun Han^4^, Tao Liu^5^, Li Jia^4^, Fei Gao^4^, Xiaojun Liu^4^, Mi Chen^4^, Guangming Yi^4^, Hongchun Dai^4^, Jun He^4^

**1** Department of Clinical Medicine, Southwest Medical University, Luzhou, China, **2** Department of Gastroenterology, The Third Hospital of Mianyang (Sichuan Mental Health Center), Mianyang, China, **3** Department of Radiotherapy, The Fourth Hospital of Hebei Medical University, Shijiazhuang, China, **4** Department of Oncology, The Third Hospital of Mianyang (Sichuan Mental Health Center), Mianyang, China, **5** Department of Oncology, The First Affiliated Hospital of Chengdu Medical College, Chengdu, China
